# Predictors of quality of life for autistic adults

**DOI:** 10.1002/aur.1965

**Published:** 2018-05-07

**Authors:** David Mason, Helen McConachie, Deborah Garland, Alex Petrou, Jacqui Rodgers, Jeremy R. Parr

**Affiliations:** ^1^ Institute of Health and Society, Newcastle University Newcastle upon Tyne UK; ^2^ National Autistic Society Newcastle upon Tyne UK; ^3^ Institute of Neuroscience, Newcastle University Newcastle upon Tyne UK; ^4^ Complex Neurodevelopmental Disorders Service, Northumberland Tyne and Wear NHS Foundation Trust Newcastle upon Tyne UK

**Keywords:** autism, quality of life, public mental health, gender

## Abstract

Research with adults on the autism spectrum is as yet limited in scope and quality. The present study describes quality of life (QoL) of a large sample of autistic adults in the UK and investigates characteristics that may be predictive of QoL. A total of 370 autistic adults from the Adult Autism Spectrum Cohort‐UK (ASC‐UK) completed the WHOQoL‐BREF, and the Social Responsiveness Scale (SRS, autism symptom severity), along with the ASC‐UK registration questionnaire giving information on mental health and their life situation. QoL for autistic adults was lower than for the general population for each WHOQoL domain. Younger participants reported higher QoL than older participants in psychological and environment domains. Males reported higher physical QoL than females, and females reported higher social QoL than males. Significant positive predictors of QoL were: being employed (physical QoL), receiving support (social and environment QoL), and being in a relationship (social QoL). Having a mental health condition and higher SRS total score were negative predictors of QoL across all four domains. Autistic adults require access to effective mental health interventions, and informal and formal support for their social difficulties, to improve their quality of life. ***Autism Res*** *2018, 11: 1138–1147*. © 2018 The Authors Autism Research published by International Society for Autism Research and Wiley Periodicals, Inc.

**Lay summary:**

There has been limited research into the lived experience of autistic adults. Using the World Health Organization quality of life measure, we found that autistic people (370) in the UK reported their quality of life to be lower than that of the general population. Better quality of life was associated with being in a relationship; those with a mental health condition had poorer quality of life. This research suggests some ways in which autistic people can be helped to improve their quality of life.

## Introduction

Autism spectrum disorders (ASD) have been widely researched in children; however, the quantity and quality of adult research, including research with those diagnosed as adults, lags behind research conducted with autistic children [Brugha et al., 2015; Gotham et al., 2015]. Much of the current research evidence concerning autistic adults is limited by small sample sizes and most participants being ‘high functioning’ [Levy & Perry, 2011].

This study focuses on the quality of life (QoL) of autistic adults. The World Health Organization (WHO) defines QoL as ‘[an] individual's perceptions of their position in life in the context of the culture and value systems in which they live and in relation to their goals, expectations, standards and concerns’ [Harper, 1998]. This definition is, therefore, a subjective appraisal of how the individual relates to the world in the context they currently experience. The WHO's operationalization of QoL is summarized into four domains: physical (including pain, energy), psychological (including positive and negative feelings, concentration), social (personal relationships, friendships, and sex life), and environment (including monetary resources, safety, partaking in leisure activities) [Harper, 1998].

What do we know about the QoL of autistic adults, and what may be the factors which predict better QoL in this population? The reasons to explore these questions include being able to suggest appropriate targets for interventions and services, or to identify individuals who require additional support. Burgess and Gutstein [2007] were the first to review studies on QoL in autistic individuals. Compared to normative samples, autistic adults on average report significantly lower subjective QoL [Jennes‐Coussens, Magill‐Evans, & Koning, 2006; Kamp‐Becker, Schröder, Remschmidt, & Bachmann, 2010; Khanna, Jariwala‐Parikh, West‐Strum, & Mahabaleshwarkar, 2014; Van Heijst & Geurts, 2015]. However, many studies are small and potentially not representative, and larger more inclusive samples are required to accurately assess QoL for autistic people [Ayres et al., 2017].

A number of potential predictors of QoL have been investigated. A recent meta‐analysis of potential predictors of, and factors relating to QoL, including studies with both self and proxy report, found no relationship with age in the subjective QoL of autistic adults, which the authors comment may be due to a lack of studies including older autistic individuals [Van Heijst & Geurts, 2015]. In the general population, there is evidence of aging effects on QoL when measured using the World Health Organization measure (WHOQoL‐BREF). Skevington and McCrate [2012] found age was a greater predictor of QoL than other demographic variables, with those in their 20s and 30s reporting better QoL than those in their 60s and 70s.

Gender effects have also been found; Kamio and colleagues found that autistic males reported significantly higher psychological and social QoL compared to autistic females [Kamio, Inada, & Koyama, 2013]. This gender effect has not been investigated in other studies using the WHOQoL‐BREF and so the present study sought to contribute to the understanding of gender and age differences in QoL in a large varied sample of autistic people.

Other predictors of higher QoL in autistic adults in previous studies have been varied and somewhat contradictory. They include perceived informal support, but not levels of formal support or autism severity [assessed using the QoL‐Questionnaire, a QoL measure for persons with intellectual disabilities, Renty & Roeyers, 2006]. For older autistic people (aged 53–83), van Heijst and Geurts did find that greater autism severity was associated with lower QoL [assessed using the SF‐36, a health‐related QoL questionnaire, Van Heijst & Geurts, 2015]. Social and psychological predictors of QoL include extraversion and not having been bullied [assessed with the WHOQoL‐BREF, Hong et al., 2016]; social support and coping strategies [using the SF‐12, a short version of the SF‐36, Khanna et al., 2014]; and having employment and living independently [assessed with the WHOQoL‐BREF, Moss et al., 2017]. Kamio and colleagues identified a supportive mother, early diagnosis, and lack of aggressive behavior as significantly predicting better Social and Psychological QoL domains of the WHOQoL‐BREF [Kamio et al., 2013]. Given that the literature reports a range of predictors, several different measures of IQ, and some contradictory findings, the present study aims to add to current knowledge by clarifying the strongest predictors in a large sample using one well‐validated measure.

A variety of questionnaires have been used to measure QoL in research with autistic individuals [Ayres et al., 2017]; the WHOQoL‐BREF has been used the most frequently [Harper, 1998; Skevington, Lotfy, & O'Connell, 2004]. Of the four QoL domains: physical health, psychological, social relationships, and environment, social QoL is most commonly reported the lowest of the four in autistic adults [Jennes‐Coussens et al., 2006; Kamp‐Becker et al., 2010; Lin, 2014] and environment QoL most often reported the closest to normative levels [Hong et al., 2016; Jennes‐Coussens et al., 2006; Lin, 2014; Moss et al., 2017]. The WHOQoL‐BREF has been shown to have reliability and validity in some relevant samples (such as proxy report of individuals with intellectual disability) [Mugno, Ruta, D'Arrigo, & Mazzone, 2007; Oliver et al., 2005].

## Aims

(1) To consider the self‐reported QoL of a large sample of autistic adults in relation to UK norms. (2) To investigate potential predictors of QoL including demographic and social characteristics, and mental and physical health conditions, with a view to identifying risk factors that could inform interventions and services.

## Method

### Participants

Data were from an ongoing longitudinal study into the life experiences of autistic adults, the Adult Autism Spectrum Cohort‐UK (ASC‐UK; http://www.autismspectrum-uk.com/). The ASC‐UK project at Newcastle University recruits participants from a diverse range of UK sources. In this sample of 370 autistic people, 155 (41.9%) were recruited through NHS autism diagnostic services, 52 (14.1%) through voluntary organizations, 136 (36.7%) through social media, and word of mouth, and 27 (7.3%) did not indicate how they were recruited. All participants complete a general autism and other characteristics/demographics questionnaire (the ASC‐UK registration questionnaire), the WHOQoL‐BREF, and Social Responsiveness Scale adult self‐report (SRS). For participants who lack capacity to consent for themselves, a relative/carer formally makes that judgment and completes questionnaires on behalf of the individual. When signing up to the ASC‐UK study, participants inform the research team how they wish to be contacted. Therefore, each measure is provided to participants either electronically or on paper as they prefer.

### Ethics

A favorable ethical opinion for this study was granted by Wales Research Ethics Committee 6 (reference–16/WA/0295).

### Measures

***ASC‐UK registration questionnaire***. This measure comprises 78 items, to collect demographic and characterization data across 10 domains of the participant's life: diagnosis including autism, autism spectrum disorder, Asperger syndrome; everyday life including relationship status; home life including living alone or with family members (family of origin or spouse/partner); employment including paid employment, volunteering, or retired; education including type of school and qualifications achieved; support including who supports the adult and how often support is needed; mental health/neurological conditions including current diagnoses and type of medication/therapy; physical health conditions; and autism spectrum in other family members. The question and response options were designed in consultation with the autism community for comprehensiveness and clarity. (See Supporting Information Table S1 for more information on response options and categorization of example questions.)

***WHOQoL‐BRE*.** The WHOQoL‐BREF [Harper, 1998] has 26 items, two general and the rest reported in four QoL subscales: physical (seven items, e.g., pain, sleep, energy levels), psychological (six items, e.g., happiness, sense of self, concentration), social (three items, e.g., relationships, sex life), and environment (eight items, e.g., financial status, access to health services, safety, transport). Each question is rated on a five‐point Likert scale for ‘how much’, ‘how often’, ‘how good’, or ‘how satisfied’ they have felt in the past 2 weeks. Participant data were cleaned and calculated as indicated in the WHOQoL‐BREF manual, for example, excluded if more than 20% missing data (*n* = 2). Raw scores for each subscale were transformed into standardized scores; higher scores indicate better QoL.

***Social Responsiveness Scale Adult (self‐report)*.** The SRS [Constantino & Gruber, 2012] measures autism characteristics for participants aged 19 years old and above. It consists of 65 items and utilizes a four‐point Likert scale from ‘not true’ to ‘almost always true’. The data are transformed to a ‘0’ to ‘3’ Likert scale generating a score from 0 to 195. The questions focus on behavior over the past 6 months. Higher scores on the SRS indicate more severe social communication impairment.

### Data analysis

Data were analyzed using SPSS version 22.0. Initial exploration of the data revealed that 0–4.5% of responses were missing across the 26 items of the WHOQoL‐BREF. Little's Missing Completely At Random (MCAR) test was computed for all 26 items. The test was nonsignificant (χ^2^ = 144.23, df = 124, *P* = .103), and therefore expectation maximization was used to impute the missing data points [Myers, 2011].

MANOVA was used to investigate the effect of age and gender on the subscales of the WHOQoL‐BREF. Significant effects were further investigated by using a series of one‐way ANOVAs for each relevant factor (age or gender).

Multiple hierarchical regression was used to identify significant predictors of QoL subscales. Age, gender, age at diagnosis, and relevant demographic data were entered in two blocks as detailed below. Categorical demographic variables were dummy coded.

The MANOVA and regression analyses were calculated twice, first, with the entire sample and then excluding the adults who did not report a formal diagnosis. For the MANOVA, the pattern of results was not at all different and so the data are presented for the full sample. For the regression analysis, there were some minor differences in terms of which variables were significant predictors for the first block, but almost none for the full model. See Supporting Information Table S2 for the regression analysis for those who report a formal diagnosis only.

## Results

Data were available on 370 participants. Table 1 gives the ages and proportions of each gender. For the autistic people who gave consent themselves, 78.4% completed the measures unaided, and 19.6% completed them with help including 18 adults who had a proxy responder (2% did not answer the question).

**Table 1:** Participants’ Demographic Information

Characteristic	Range	Mean	SD
Age (years)	17–80	41.61	± 14.42
Male	17–74	43.03	± 15.53
Female	18–80	40.59	± 12.87
Age at diagnosis (years)	2–74	36.89	17.12
Male	2–74	37.04	18.67
Female	3–69	37.76	14.50

	*N*	%
Gender		
Male	199	53.8
Female	158	42.7
Other/Rather not say/not reported^a^	13	3.5
Capacity to self‐report		
Capable of self‐report	352	95.1
Lacking capacity	18	4.9
Current relationship status		
Currently in a relationship	151	40.8
Currently single	196	53.0
Other/not reported	23	6.2
Current living status		
Live with a family member	122	33.0
Do not live with a family member	241	65.1
Other/not reported	7	1.9
Support^b^ received		
Yes	186	50.3
No	159	43.0
Not reported	25	6.8
Current employment status		
Employed (inc. self‐employed)	135	36.5
Other^c^/not reported	235	63.5
Educational qualifications		
Higher^d^ education qualifications	154	41.6
Other/not reported	216	58.4
Current mental health condition diagnosis		
Yes	262	70.8%
No	81	21.9%
Not reported	27	7.3%
Current physical health condition diagnosis		
Yes	260	70.3%
No	84	22.7%
Not reported	26	7.0%

a Note that these participants are excluded from all subsequent analyses which include gender; ^b^data are collected about support in the home with daily living tasks, help at work, that is interacting with co‐workers, managing money, organizing a diary or planning daily activities; ^c^‘other’ includes being a career for a relative, long‐term illness, being a full time student, or unable to work; ^d^‘higher education' is first‐degree level qualification or above.

Of the 370 participants, 66 (18%) were aged 17–25 years, 114 (31%) participants aged 26–40, 150 (41%) participants aged 41–60, and 38 (10%) participants aged 61 and above. Two participants did not complete the age question. Seventy percent reported a current diagnosis of a mental health condition (most commonly depression and/or anxiety). Seventy percent reported a current physical health condition (e.g., sleep problems, or hypertension) (see Table 1). The mean SRS score was 111.65 (SD = 28.49). All but seven had a total score above cut‐off for “social impairment” (i.e., 52, ref). There was no significant gender difference for SRS (males mean score 110.63, SD = 27.88; females mean score 113.86, SD = 28.31; *t*(314) = −1.019, *P* = .309).

Of those who reported age at diagnosis (337), the majority received diagnosis in adulthood (84.6%); 15.4% were diagnosed in childhood. The data set included 31 participants who stated either that they were awaiting diagnostic assessment, or that they suspected they were on the autism spectrum. Differences in autism severity, age and gender from the rest of the sample were tested. Those without a formal diagnosis reported a lower mean SRS total score than those with a formal diagnosis (99.73, SD = 29.87 vs. 113.22, SD = 27.67; *t*(314)=–2.405, *P* = .017). However, the mean for those without a formal diagnosis was still well above the cut‐off of 52, [Constantino & Gruber, 2012]. There were no significant differences in terms of age grouping (χ^2^ (df = 3, *N* = 357) = 6.705, *P* = .082) or gender (χ^2^ (df = 1, *N* = 357)= .744, *P* = .388). Therefore, the 31 without a formal ASD diagnosis were retained in the descriptive analyses.

### Descriptive statistics

Table 2 presents QoL data from the current study and data collected from UK participants to validate the WHOQoL‐BREF as a clinical measure suitable for individual assessment (data are from ‘well’ participants, rather than those with different illness diagnoses; *n* = 1324–1328) [Skevington & McCrate, 2012]. Reported QoL for autistic adults was lower across all four domains than UK norms. The UK data were compiled from numerous research sites, and the primary data were not available for formal comparison; Cohen's *d* was computed for each subscale showing moderate to large differences (see Table 2).

**Table 2:** Means and Standard Deviations for WHOQoL‐BREF Subscales and Normative Data for the UK Population

Group	Mean physical (SD)	Mean psychological (SD)	Mean social (SD)	Mean environment (SD)
ASC‐UK	47.95 (18.83)	45.74 (16.87)	40.24 (21.99)	55.53 (19.95)
ASC‐UK^a^	49.22 (18.41)	44.38 (17.81)	40.32 (22.32)	54.81 (20.00)
UK norms^b^	76.49 (16.19)	67.82 (15.56)	70.52 (20.67)	68.20 (13.81)
Cohen's d	1.63	1.36	1.42	0.74

a Excluding those without a formal diagnosis; ^b^taken from normative data (Skevington & McCrate, 2012) (‘well’ participants; *n* = 1324–1328); *d* = 0.2 considered a small effect, 0.5 a medium effect, and 0.8 a large effect.

In order to investigate possible differences in QoL between males and females, and between age groups, a 4 × 2 × 4 MANOVA was calculated. Table 3 presents the results of the MANOVA and subsequent ANOVAs (one‐way ANOVA of age on each subscale score and gender on each subscale) with partial *η* ^2^ as a measure of effect size.

**Table 3:** MANOVA Results for Age, Gender, and WHOQoL‐BREF Domains

Statistic	Wilk's λ	F	*P*	Partial η^2^
MANOVA				
Age × gender	0.947	1.58	0.091	0.02
Age	0.910	2.75	0.001	0.03
Gender	0.956	3.97	0.004	0.04
ANOVA—age				
Physical	–^a^	1.10	0.347	0.01
Psychological	–^a^	3.95	0.009	0.03
Social	–^a^	1.83	0.141	0.02
Environment	–^a^	3.67	0.012	0.03
ANOVA – gender				
Physical	–^a^	5.56	0.019	0.02
Psychological	–^a^	1.63	0.202	0.01
Social	–^a^	4.12	0.043	0.01
Environment	–^a^	3.12	0.078	0.01

a Wilk's λ is not applicable to univariate tests. Partial *η* ^2^ = 0.01 for a small effect, 0.06 for a medium effect, and 0.13 for a large effect.

**Figure 1:**
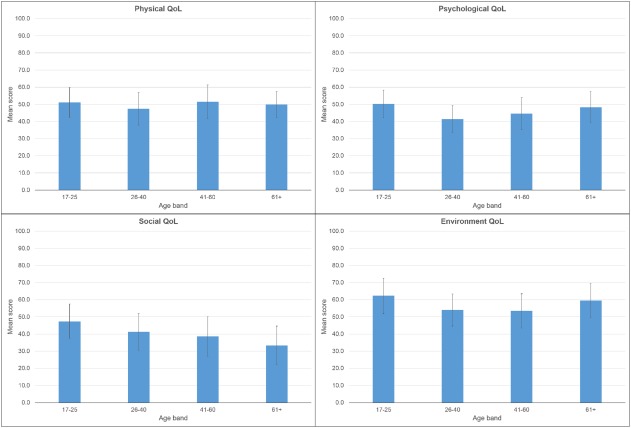
Mean scores for subscales of the WHOQoL‐BREF (scored from 0 to 100) by age band (in years) with errors bars showing one standard deviation.

Tukey post‐hoc comparisons were computed for the four age groupings on the psychological and environment subscales. For psychological QoL, the age 17–25 years group (mean = 50.23, SD = 16.06) reported higher QoL than the 26–40 years group (mean = 41.27, SD = 15.76; *P* = .044). For environment QoL, the 17–25 group (mean = 62.28, SD = 20.66) reported higher QoL than the next two higher age groupings: 26–40 group (mean = 53.78, SD = 18.76; *P* = .042) and the 41–60 group (mean = 53.54, SD = 20.02; *P* = .018) (see Fig. 1). However, the effect sizes were small.

Gender showed a main effect on both the physical and social subscales. Males reported higher physical QoL than females (mean = 52.98, SD = 17.32 vs. mean = 45.98, SD = 19.57, respectively), and females reported higher social QoL than males (mean = 42.52, SD = 22.53 vs. mean = 38.62, SD = 22.11, respectively). However, the effect sizes were small.

### Other predictors of QoL

Hierarchical regression analysis was used to explore other potential predictors of QoL. Age, gender, and age at diagnosis were entered into the first block of the regression analysis (model 1). The second block comprised the following categorical variables (model 2): relationship status (in a relationship versus single), living status (living with family members versus alone or other arrangement), being in independent employment (versus being unemployed, retired, training or supported employment), having a current diagnosed mental health condition, having a current diagnosed physical health condition, receiving external support (with health or finances), and having higher education qualifications. The SRS total score was also added into the second block.

Model 1 explained 3.8%, 5.2% and 4.7%, respectively, of the variance for the domains of physical QoL, psychological QoL, and environment QoL. Being female significantly predicted lower physical QoL (β = −0.18, *P* < .05), lower psychological QoL (β = −0.20, *P* < .01), and lower environmental QoL (β = −0.20, *P* < .01). Model 1 did not significantly predict social QoL (*P* = .07). Age at diagnosis did not significantly explain the variance of any QoL domain.

The additional categorical variables differentially predicted QoL across the four domains. This, in turn, increased the variance explained by model 2 for each QoL domain (see Table 4 and Fig. 1). For physical QoL, four variables explained 39.6% of the variance (*R* ^2^ = .396, *F*(11,252)=14.335, *P* < .001). Employment positively predicted QoL; being female, having a mental health diagnosis, and higher SRS total predicted lower QoL. For psychological QoL, three variables explained 32.3% of the variance (*R* ^2^ =.323, *F*(11,252)=10.436, *P* < .001). Being female, having a mental health diagnosis, and higher SRS total predicted lower QoL; none of the additional variables positively predicted QoL scores for this domain. For social QoL, six variables explained 24.6% of the variance (*R* ^2^=.246, *F*(11, 252) = 7.135, *P* < .001). Being in a relationship, receiving support, and having a higher level of education significantly predicted higher scores in this domain; being older, having a mental health diagnosis, and higher SRS total predicted lower QoL. For environment QoL, four variables explained 38.3% of the variance (*R* ^2^ = .383, *F*(11,252) = 13.587, *P* < .001). Receiving support significantly predicted higher QoL scores in this domain; being female, having a mental health diagnosis, and higher SRS total predicted lower QoL. Having a physical health condition, living independently, and age at diagnosis were not significant predictors of QoL in model 2. When the regression analysis was restricted to those with a formal diagnosis (see Supporting Information Table S2), later age at diagnosis was a negative predictor of psychological and environment QoL, but this finding was not significant in the full regression model.

**Table 4:** *R* ^2^, Standardized β Coefficients for the Positive and Negative Predictors, and Model Significance for Each Subscale of the WHOQoL‐BREF

Subscale predictors	*R* ^2^	*P*	Β			
			Positive predictors of QoL		Negative predictors of QoL	
Physical						
Model 1^a^	0.038	.021	–	–	Being female	−0.181^*^
Model 2^b^	0.396	<.001	Being employed	0.111^*^	Being female Mental health diagnosis SRS total	–0.130^*^ –0.211*** –0.414***
Psychological					–	
Model 1^a^	0.052	.004	–	–	Being female	−0.199**
Model 2^b^	0.323	<.001	–	–	Being female Mental health diagnosis SRS total	–0.157** –0.272*** –0.375***
Social						
Model 1^a^	0.028	.072	–	‐	Age	−0.150^*^
Model 2^b^	0.246	<.001	Being in a relationship Receiving support Higher education	0.296*** 0.128^*^ 0.135^*^	Age Mental health diagnosis SRS total	–0.182** –0.196** –0.263***
Environment						
Model 1^a^	0.047	.014	–	‐	Being female	−0.195**
Model 2^b^	0.383	<.001	Receiving support	0.180**	Being female Mental health diagnosis SRS total	–0.163** –0.249*** –0.441***

Predictors were age, age at diagnosis, and gender; ^b^predictors were relationship status, living status, being employed, receiving external support, education level, having a diagnosed mental health condition, having a diagnosed physical health condition, and SRS total.

*P < .05, **P < .01, ***P < .001

## Discussion

This is the largest study to describe the subjective QoL of people on the autism spectrum using the WHOQoL‐BREF and confirms the findings of previous smaller studies that QoL is generally lower for people on the autism spectrum compared to the general population.

The sample is similar in characteristics to a number of recent studies of autistic adults in USA and Europe. In the present sample, 33% lived with family (family of origin or spouse or partner) comparable to Bishop‐Fitzpatrick et al. [2016], *n* = 18, who report 44% for their sample; 36.5% were competitively employed for 10 hr or more per week which is similar to Helles, Gillberg, Gillberg, and Billstedt [2017], *n* = 50, who report employment rates of 40%; 41.6% had a Bachelor's degree or higher which is a similar proportion to the 42% reported by Gotham et al. [2015], *N* = 254. A current diagnosis of a least one mental health condition was reported by 70.8% of the present sample, midway between the 50% reported by Helles et al. [2017] and the 86% reported in Gotham et al. [2015]. The present study contained a large proportion of females (*n* = 158, 42.7%) rather than the 4:1 male:female ratio expected from epidemiological studies of children. The majority of the sample did not need help filling in the measures (78.4%), and so the sample comprised mostly relatively able autistic adults who were mainly diagnosed in adulthood (mean age at diagnosis = 36.9). Thus, the findings of this study concerning predictors of QoL are likely to be comparable to many other studies of autistic adults, and generalizable to autistic adults who can self‐report; as ASC‐UK aims to recruit greater proportions of adults with intellectual disability and those diagnosed as children, the strength of the conclusions can be tested further.

Three main characteristics were predictive of lower QoL in almost all domains: being female, having a current mental health diagnosis and higher severity of autism symptoms. A number of factors positively predicted QoL: better physical QoL was predicted by being employed, greater social QoL was predicted by being in a relationship and receiving support, and environment QoL was also predicted by receiving support. These results provide evidence‐based indications of specific potential targets for interventions and services to improve QoL for autistic people; for example, older autistic women with mental health conditions are one group particularly likely to need support through services. It is, however, important to note that our findings are cross‐sectional. It may be the case that, for example, those who report higher social QoL are more able to initiate and maintain a relationship with a partner and/or achieve higher education attainment. Therefore, our results do not support a causal interpretation between our predictor variables and QoL scores.

The predictors identified here are in line with some but not all findings of earlier research. For example, Kamio et al. [2013] found QoL to be significantly lower for females, though that study reported only on the social and psychological domains. In contrast, in the present study there was a small difference suggesting females had higher satisfaction with their social QoL than males. A recent qualitative study has suggested that autistic females are more socially motivated and able to maintain friendships than males [Bargiela, Steward, & Mandy, 2016]. However, Hull et al. [2017] have noted that ‘camouflaging’ by autistic people is described as exhausting, and in the long term can be a threat to self‐perception. These findings suggest that females may have an advantage in social situations, and males may find social situations more challenging, but future work is needed to further explore the mechanisms of gender differences in QoL.

The psychological QoL scale includes items related to being satisfied with oneself, the presence/absence of mental health conditions, and having a meaningful life. Mental health conditions are more prevalent for individuals on the autism spectrum [Gillberg, Helles, Billstedt, & Gillberg, 2016] with the chances of having at least one mental health condition ranging from 70% to 79% [Lever & Geurts, 2016; Perkins & Berkman, 2012]; however, some studies have found rates to be lower [Gotham et al., 2015] and overall mental health data for this population can be highly variable [Howlin & Magiati, 2017]. In relation to the present sample, participants may have been diagnosed with other mental health conditions prior to their autism diagnosis and therefore have received less than optimal support for their mental health difficulties [Perkins & Berkman, 2012]. Employment is widely reported to underpin a range of QoL components in the typically developing population (i.e., better mental health, life satisfaction, marital/family satisfaction) and helps facilitate economic self‐sufficiency and social inclusion [Walsh, Lydon, & Healy, 2014]. The present finding that being independently employed was a positive predictor of physical QoL in autistic people is, therefore, unsurprising, especially in light of the domain's questions which ask about energy levels, mobility, physical pain, and capacity for work. As mentioned above, we are not suggesting a direction to the association; it could well be that those who report higher physical QoL are more able to secure and maintain employment. Other QoL domains (such as social QoL) might have been predicted had the analysis focused on unemployment as the reference group. Rates of unemployment are high in the autism population [Roux et al., 2013] and can be significantly improved through customized training programs that may lead to competitive employment [Wehman et al., 2016].

The significant effects of age have not been reported by previous smaller studies. Some effects of aging may not be specific to autistic people; the present age‐related findings do agree with a large general population study using the WHOQoL‐BREF. Skevington and McCrate [2012] found reduced QoL at older ages (findings not reported by subscale); the present study found age to be a negative predictor of Social QoL. Similarly, being younger was associated with higher psychological and environment QoL. Services are still not able to fully meet the needs of autistic adults [Howlin, Alcock, & Burkin, 2005] which would suggest that lower QoL for older adults in part reflects inadequate access to appropriate services. These types of support can be formal (i.e., from services) or informal, but having it available can make an important difference and avoid later escalation of problems [National Institute for Health and Clinical Excellence, 2012]. This is an important consideration given daily living skills (personal care, housekeeping, meal preparation, etc.) have been shown to plateau during late 20s and show a further decline during mid 30s for some autistic adults [Smith, Maenner, & Seltzer, 2012]. While autistic adults may be more able to cope with social isolation in older age [Happé & Charlton, 2011], the loss of perceived informal support (having someone to talk to, someone to do things with) is a significant predictor of lower QoL [Renty & Roeyers, 2006]. Our findings showed being in a relationship and receiving support were positive predictors of social QoL. Therefore, lower social QoL for older autistic adults could reflect either increased physical and mental health conditions as people age [Happé & Charlton, 2011] or a loss of supportive people (e.g., due to death of family members). The effects are likely to be exacerbated in those individuals with more severe autism symptoms, such as lack of flexibility and reluctance to try new social situations.

### Strengths/Limitations

This study has many strengths and some limitations. Strengths included the UK‐wide sampling frame with participants joining the ASC‐UK cohort from a range of sources. The large sample gave power to undertake analyses by age and gender. A robust measure of QoL is a strength, increasing the validity of the analysis; furthermore, the WHOQoL‐BREF has been further validated as a QoL measure for the autism population [McConachie et al., 2017] which strengthens the present findings.

Questionnaire data completeness was excellent, likely to be due in part to the extensive consultation with the autism community about the methods and materials used in the ASC‐UK study [Parr, 2016]. Many adults want to take part in research that enables them to tell others about their lives with a view to identifying support strategies for the autism community for the future.

There were some limitations of the study. The demographic data entered as predictors of QoL to some extent lacked specificity. For example, information on the type and extent of ‘received support’ from the registration questionnaire was limited, which may affect discriminatory power. The groupings adopted for analysis might have been considered differently; for example, we took ‘independent employment’ as the reference group, but could have chosen to combine this with supported employment and/or volunteering. Future analysis can identify what are the key aspects of daily occupational activity for autistic people. The data did not permit corroboration of participants’ report of variables such as having a current mental health diagnosis, nor was there formal confirmation of diagnosis. The data are cross‐sectional, limiting interpretation of causal relationships. As the ASC‐UK study continues, the capacity to undertake analyses relating to QoL and changing circumstances will emerge.

A further possible limitation involves inclusion of responses on behalf of individuals unable to complete the questionnaire for themselves (<5%). Given the subjective nature of QoL it may be, prima facie, that proxy‐reports cannot be a valid substitute for a first person rating. However, adults with intellectual disability are frequently excluded from research due to issues with obtaining valid informed consent [Hamilton et al., 2017]. As such, this excludes a portion of the autism community from being recognized in QoL research. Further, Hong and colleagues found quite high correlations between adult self‐report and maternal proxy report [“how she thinks her adult child with ASD feels about his/her own QoL”, Hong et al., 2016, p. 1370]) and this type of report was more significantly related to the adult self‐report than maternal report (i.e., the mother's own perceptions). Therefore, we included participants who lack capacity to respond for themselves. As the ASC‐UK study continues, it will be possible to analyze these data separately and validate them against appropriately adapted qualitative interviews.

### Implications

The study findings are that autistic people on average have lower QoL than the general population in the UK; however, analysis of predictors has given some indications to guide where supportive services might intervene. A recent systematic review of studies of adult outcomes found that fewer than 20% of autistic people enjoy ‘good’ outcomes (defined objectively as independent living, friendships, and employment) and almost 50% have poor or very poor outcomes by these criteria [Steinhausen, Mohr Jensen, & Lauritsen, 2016]. Although there is growing awareness of the need to develop diagnostic and intervention services for autistic adults, with policy initiatives including the Adult Autism Strategy [Department of Health, 2015] in UK, there is little basis for identifying the most vulnerable groups and priorities for action. What is clear are the difficulties experienced by autistic people in accessing appropriate support. Intervention services for autistic adults are limited [Shattuck et al., 2012]; for example, there is a lack of adequate transition (i.e., school to work) programs and co‐ordination between support services [Gerhardt & Lainer, 2011]. However, third sector organizations (i.e., voluntary or community organizations, or registered charities) are trying to deliver a range of initiatives, such as promoting positive lifestyle and skills training relevant to the needs of autistic adults [Guldberg, Mackness, Makriyannis, & Tait, 2013]. Our study shows that those individuals with higher severity of autism characteristics and a current mental health diagnosis are particularly vulnerable to low QoL, as are autistic women. Those who are receiving support in their lives—such as in the home with daily living tasks, help at work including how to interact with co‐workers, help with managing money, organizing a diary or planning daily activities—report higher social and environment QoL. Longitudinal studies would help further to describe the QoL of autistic adults over time in order to identify points for intervention.

## Authors’ Contributions

H.M., D.G., and J.P.R. conceived and designed the study. A.P. collected the data, D.M. analyzed the data, with supervision from H.M. and J.R. All authors were involved in the interpretation of the data. D.M. drafted the report, which was reviewed by all authors.

## Conflict of Interest

The authors declare no conflicts of interest.
